# Structural basis for the inhibition of voltage-dependent K^+^ channel by gating modifier toxin

**DOI:** 10.1038/srep14226

**Published:** 2015-09-18

**Authors:** Shin-ichiro Ozawa, Tomomi Kimura, Tomohiro Nozaki, Hitomi Harada, Ichio Shimada, Masanori Osawa

**Affiliations:** 1 Graduate School of Pharmaceutical Sciences, The University of Tokyo, Hongo, Bunkyo-ku, Tokyo 113-0033, Japan; 2 Keio University Faculty of Pharmacy, Shibakoen, Minato-ku, Tokyo 105-8512, Japan

## Abstract

Voltage-dependent K^+^ (K_v_) channels play crucial roles in nerve and muscle action potentials. Voltage-sensing domains (VSDs) of K_v_ channels sense changes in the transmembrane potential, regulating the K^+^-permeability across the membrane. Gating modifier toxins, which have been used for the functional analyses of K_v_ channels, inhibit K_v_ channels by binding to VSD. However, the structural basis for the inhibition remains elusive. Here, fluorescence and NMR analyses of the interaction between VSD derived from K_v_AP channel and its gating modifier toxin, VSTx1, indicate that VSTx1 recognizes VSD under depolarized condition. We identified the VSD-binding residues of VSTx1 and their proximal residues of VSD by the cross-saturation (CS) and amino acid selective CS experiments, which enabled to build a docking model of the complex. These results provide structural basis for the specific binding and inhibition of K_v_ channels by gating modifier toxins.

Voltage-dependent K^+^ (K_v_) channels alter their K^+^-permeability across membrane lipid bilayers in a membrane potential-dependent manner, playing crucial roles in nerve and muscle action potentials^1^. The K_v_ channels function as a tetramer, in which each subunit possesses six transmembrane helices, S1–S6. Tetrameric assembly of the S5–S6 regions of the four subunits (referred to as a pore domain) forms a pore for the K^+^-permeation, in which a crossing of the four S6 helices at the intracellular side of the pore (referred to as a helix bundle crossing) acts as a gate to physically preclude the K^+^-permeation. The opening and closing of the gate (gating) is allosterically regulated by voltage-sensing domains (VSDs) comprised of the S1–S4 helices that are located at the periphery of the pore domain^2^,^3^. A number of positively charged residues of S4 are responsible for the membrane potential-dependent S4 shift^4^,^5^,^6^,^7^,^8^,^9^,^10^; at the resting potential, S4 shifts to the intracellular side of the membrane (‘down’ conformation), whereas during depolarization, S4 shifts to the extracellular side (‘up’ conformation). This voltage-dependent conformational change of VSD is assumed to cause the gating.

To date, a variety of peptide toxins that inhibits specific K_v_ channels have been isolated from venomous organisms such as snakes, scorpions and spiders, and used for the characterization of the K_v_ functions^11^. These toxins can be classified into two groups, a pore blocking toxin and a gating modifier toxin. Pore blocking toxins target the extracellular side of the pore domain, and the structural basis, on which the toxins physically occlude the pore, has been revealed^12^,^13^,^14^,^15^. On the other hand, gating modifier toxins bind to VSD, and are assumed to alter the conformation and energetics of voltage-dependence of VSD^16^,^17^,^18^ whereas the structural basis for the inhibition has not been fully elucidated.

Recently, the structures of several gating modifier toxins targeting K_v_ channels such as VSTx1, SGTx1 and HaTx, have been determined^19^,^20^,^21^. These toxins commonly possess a cluster of solvent-exposed hydrophobic residues (referred to as a hydrophobic patch) surrounded by highly polar residues, enhancing the affinity for their target K_v_ channels by allowing the toxins to partition into the membrane^17^,^21^,^22^,^23^,^24^,^25^. However, mutagenic study reported that the hydrophobic patch of SGTx1 also plays a critical role in the recognition of its target, K_v_2.1, in the membrane^26^.

In addition, VSTx1 reportedly inhibits an archaebacterial K_v_ channel, K_v_AP^2^, where VSTx1 exclusively binds to the VSD and the pore domain is not required for the toxin-channel interaction^27^. Furthermore, electro physiological studies suggested that the K_v_AP is inhibited upon depolarization by recognizing the up conformation of VSD^28^. However, no structure of VSD in complex with a gating modifier toxin has been reported, and thus it remains unknown how these toxins prevent the voltage-dependent conformational change of VSD.

In this study, we performed the fluorescence and NMR analyses of the interaction of VSTx1 and VSD derived from K_v_AP, indicating that VSTx1 stabilizes the up conformation of VSD. In addition, we identified the VSD binding residues of VSTx1 and their proximal residues of VSD by the cross-saturation (CS)^29^,^30^ and amino acid selective CS (ASCS)^31^ experiments. Based on these results, we built a docking model of VSTx1 and VSD, providing the structural basis for the specific binding and the inhibitory mechanism of K_v_ channels by gating modifier toxins.

## Results

### Characterization of the prepared VSD and VSTx1 proteins

VSD from K_v_AP (residues −12 to 136, the residue numbers correspond to those in the crystal structure^32^) solubilized in n-decyl-ß-D-maltopyranoside (DM) micelles was purified to homogeneity (Supplementary Fig. S1). Size exclusion chromatography analysis indicated that the prepared VSD exists as a monomer in the DM micelles (Supplementary Fig. S1). While ^1^H-^15^N TROSY NMR spectrum of uniformly ^2^H, ^15^N-labeled VSD exhibits a good dispersion of the signals in a ^1^H-dimension, some signals are broadened, suggesting that VSD is globally folded with some residues undergoing chemical exchange (Supplementary Fig. S1).

On the other hand, VSTx1 was expressed in *E. coli* and purified to homogeneity (Supplementary Fig. S2). We assigned backbone NMR resonances of the uniformly ^13^C, ^15^N-labeled VSTx1 by a series of triple resonance experiments (Supplementary Table S1 and Supplementary Fig. S2). Chemical shift index analysis confirmed that the secondary structure of the prepared VSTx1 is consistent with the previously reported solution structure^21^ (Supplementary Fig. S2).

### Voltage-dependent conformational change of VSD and its inhibition by VSTx1

In order to investigate the voltage effect on the VSD conformations, a fluorescence-labeled VSD, in which monobromobimane (mBBr) was chemically attached to a Cys residue mutated from Val 119 that lies at the extracellular end of S4, was reconstituted into liposomes. This assay was originally developed to study H^+^ flux through V-ATPase^33^, and then applied to study voltage-dependent conformational change of H_v_1 H^+^ channel and VSD^34^. Specifically, liposomes were prepared in the presence of 150 mM K^+^ and diluted into buffer containing lower concentration of K^+^, which generated the K^+^ gradient across the membrane. By adding a K^+^ -selective ionophore, valinomycin, the K^+^ efflux produces the membrane potential (negative inside relative to outside). When the ‘up’ to ‘down’ conformational change of the S4 helix of the reconstituted VSD with its N- and C-termini inside the liposome is caused by the formation of the membrane potential, the intensity of the fluorescence derived from mBBr, which changes the environment from the one exposed to the solvent to the one buried in the membrane, would increase. On the other hand, the VSD with its N- and C- termini outside the liposome (corresponding to the opposite orientation) would experience positive voltage upon the formation of the membrane potential, where the S4 helix would remain in the up conformation, and mBBr would remain exposed to the solution. Therefore, molecules in this orientation are not supposed to contribute to the “change” in the fluorescence intensity.

**Figure 1:**
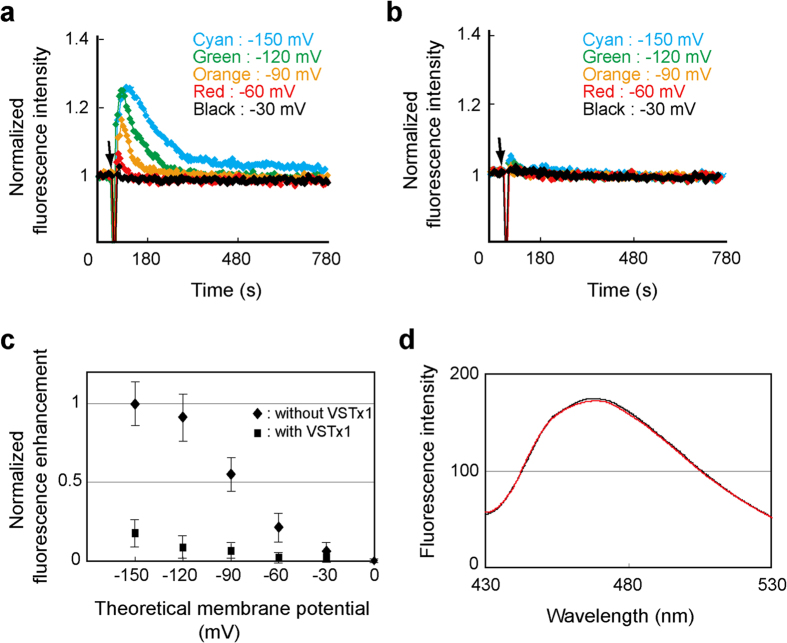
Voltage-dependent conformational change of VSD and its inhibition by VSTx1. (**a**) Relative fluorescence intensities of mBBr-labeled VSD reconstituted into liposomes in the absence of VSTx1. Valinomycin was added at the time point indicated by an arrow to form the membrane potentials as indicated. (**b**) Relative fluorescence intensity of mBBr-labeled VSD in the presence of 0.5 mM VSTx1. (**c**) The mean values of transient increases in the fluorescence plotted against the membrane potentials. Plots are normalized by the value at the theoretical membrane potential −150 mV without VSTx1. Error bars represent SD (n = 5). (**d**) Fluorescence spectra of mBBr-labeled VSD in the absence (black) and presence (red) of VSTx1.

Figure 1a shows transient increases and subsequent decays of the fluorescence intensities upon formation of the various membrane potentials, suggesting that the mBBr moiety on the extracellular end of S4 buried in the membrane transiently and returned to the initial exposed position. The baseline potential before the addition of valinomycin was confirmed to be 0 mV since the fluorescence intensity of the diluted liposomal solutions was equivalent to that of the liposomal solution suspended in the same buffer as the one used for the liposome preparation. The fluorescence decays might reflect the ‘down’ to ‘up’ conformational change of the VSD caused by the depolarization of the membrane due to the H^+^ entry through an intrinsic proton conduction pathway of VSD, as described in the previous report^34^.

Voltage-dependence of the transient fluorescent increase as shown in Fig. 1c (rhombic) suggests that VSD adopts mostly up and down conformations at 0 and −150 mV, respectively, with the half maximal value at ca. −90 mV. This half maximal value of the VSD movement was 30 to 40 mV smaller than a typical mid-point potential of the conductance-voltage curve of K_v_AP in POPE/POPG membrane^28^,^32^, which is consistent with the differences in the mid-point potentials between the VSD movements and the conductance of K_v_AP and other K_v_ channels^4^,^5^,^35^. Therefore, we conclude that the isolated VSD prepared here exhibits the voltage-dependent conformational change as previously reported.

Then, we investigated the effect of VSTx1 on the voltage-dependent fluorescent change of the mBBr-labeled VSD. Figure 1b shows the fluorescence upon the formation of the various membrane potentials in the presence of VSTx1 and Fig. 1c (square) shows the voltage-dependence of the transient fluorescence increase, clearly indicating that VSTx1 suppressed the fluorescence change of the S4-attached mBBr. Furthermore, no significant change was observed for the fluorescence profile upon addition of VSTx1 without the polarization (Fig. 1d). These results strongly suggest that VSTx1 binds to VSD in the absence of membrane potential, stabilizes the up conformation of VSD without significant structural change around S4, and inhibits the conformational change to the down conformation.

### VSD adopts an up conformation in DM micelles

**Figure 2:**
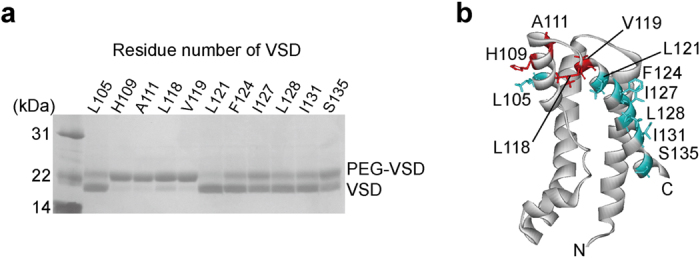
Solvent accessibilities of Cys residues introduced to the 11 different positions of VSD from S3b to S4. (**a**) Gel shift assay of VSD. The number of the residue, to which Cys was introduced, was indicated. (**b**) Mapping of the results. Highly PEGylated residues, which possess high solvent-accessibility, are colored red whereas others are cyan on the structure of VSD (PDB code 2KYH).

In order to reveal the structural basis of the inhibition of the voltage-dependent conformational change of VSD by VSTx1, we investigated the interaction between VSD and VSTx1 in the DM micelles. First, we examined whether the VSD in the DM micelles adopts an up or down conformation, based on solvent accessibilities of Cys residues introduced to the 11 positions of VSD from S3 to S4 (Fig. 2). These results indicate that water-soluble Cys modification reagent, Mal-PEG, reacted with the mutants, H109C, A111C, L118C and V119C, in which the former two mutation sites are on the C-terminal region of S3 (referred to as S3b) and the latter two are on the N-terminal region of S4. Based on the report that residues on S3b and the N-terminus of S4 are exposed to the extracellular solution in the up conformation whereas C-terminal residues of S4 are exposed to the intracellular solution in the down conformation^36^, our observations of the solvent-exposed residues are consistent with the up conformation.

### Direct binding of VSTx1 to VSD in DM micelles

**Figure 3:**
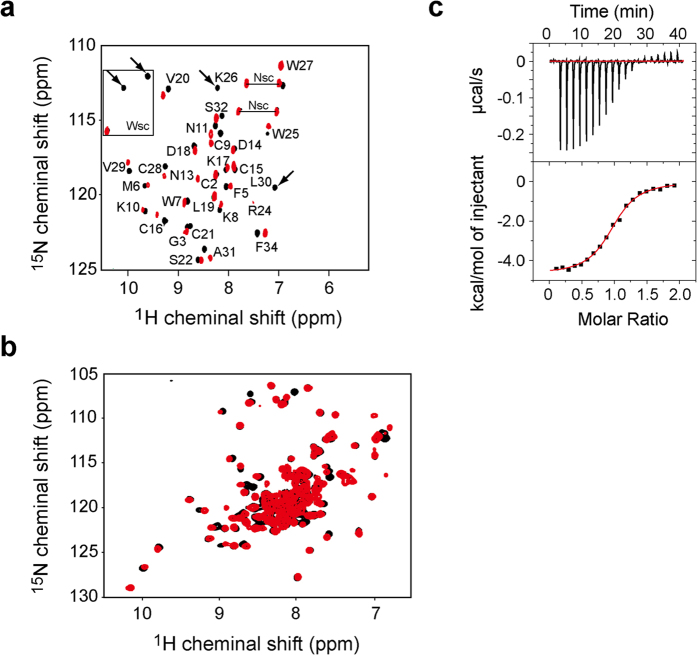
Direct interaction between VSTx1 and VSD in the DM micelles. (**a**) ^1^H-^15^N HSQC spectra of uniformly ^15^N-labeled VSTx1 in the absence (black) and presence (red) of VSD in the DM micelles. Arrows indicate the residues disappeared upon binding to VSD. “sc” indicates side chain signals. (**b**) ^1^H-^15^N TROSY spectra of uniformly ^2^H, ^15^N-labeled VSD in the absence (black) and presence (red) of VSTx1 in the DM micelles. (**c**) Isothermal titration microcalorimetric analyses of the interaction between VSTx1 and VSD in the DM micelles. Trace of the calorimetric titration of VSTx1 into the cell containing VSD (upper panel) and the integrated binding isotherm fitted using a single-site model (lower panel) are shown. The parameters obtained from the best fit are summarized; the binding stoichiometry: 0.97 ± 0.01, the dissociation constant (*K*_d_): 1.5 ± 0.2 μM, ΔH: −4.5 ± 0.2 kcal mol^−1^ and ΔS: 11.4 cal mol^−1^ K^−1^.

We investigated the interactions of VSTx1 with DM and VSD by monitoring the NMR spectra of VSTx1. First, we observed a series of ^1^H-^15^N HSQC spectra of uniformly ^15^N-labeled VSTx1 at the increasing concentrations of DM. A number of signals showed chemical shift changes, which were saturated at the DM concentration of 150 mM (Supplementary Fig. S3). Then, sequential additions of VSD into the VSTx1 solution in the presence of 150 mM DM also exhibited chemical shift changes (Fig. 3a). These results indicate the direct interactions of VSTx1 with DM and VSD. The backbone assignments of VSTx1 in complex with VSD in 150 mM DM were easily traced by sequential additions of DM up to 150 mM, followed by the additions of the VSD solution. The residue-specific analyses of the chemical shift changes of VSTx1 by DM and VSD were shown in Supplementary Figure S3.

The binding affinity between VSTx1 and VSD in the DM micelles was quantitatively analyzed by isothermal titration calorimetry (ITC). Fitting of the ITC isotherm resulted in the dissociation constant (*K*_d_) of 1.5 μM with a binding stoichiometry of 1:1 (Fig. 3c).

On the other hand, we compared ^1^H-^15^N TROSY spectra of uniformly ^2^H, ^15^N-labeled VSD in DM micelles in the presence and absence of VSTx1 (Fig. 3b). An overlay of these spectra shows that VSTx1 caused chemical shift changes larger than the half width of the signals of VSD. However, only a limited number of signals of the VSD experienced the chemical shift change upon the VSTx1 binding, suggesting that the VSD conformation is not largely affected by the VSTx1 binding. Thus, the VSD seems to remain in an up conformation even in complex with VSTx1 in DM micelles, as observed in the lipid bilayer (Fig. 1d).

It should be noted that some NMR signals of VSD are broadened as stated above. The broadening was not improved even in the presence of VSTx1, which precluded complete assignments of the NMR resonances of VSD and NOE-based structure determination of the VSTx1-VSD complex. Here, we tried to obtain structural information of the VSTx1-VSD interaction through the NMR spectra of VSTx1 reflecting the interactions with VSD and DM without the assignments of the NMR resonances of the VSD.

### DM and VSD binding residues of VSTx1 identified by the CS experiments

In order to reveal how VSTx1 recognizes VSD to inhibit K_v_AP, we applied cross-saturation (CS)^29^,^30^ and amino acid-selective cross-saturation (ASCS)^31^ methods. The former can identify VSTx1 residues binding to DM and/or VSD, and the latter can identify intermolecular proximal residue pairs between VSTx1 and VSD, both of which do not require the assignments of the NMR signals of VSD. The information on proximal residue pairs between VSTx1 and VSD enables docking of the reported structures of the two proteins since the individual structures remain essentially unchanged upon binding as suggested by the small chemical shift changes (Fig 3a,b and Supplementary Fig. S3). As the first step of this strategy for the structure determination of the VSTx1-VSD complex, we identified DM and VSD binding residues of VSTx1 by the CS method.

**Figure 4:**
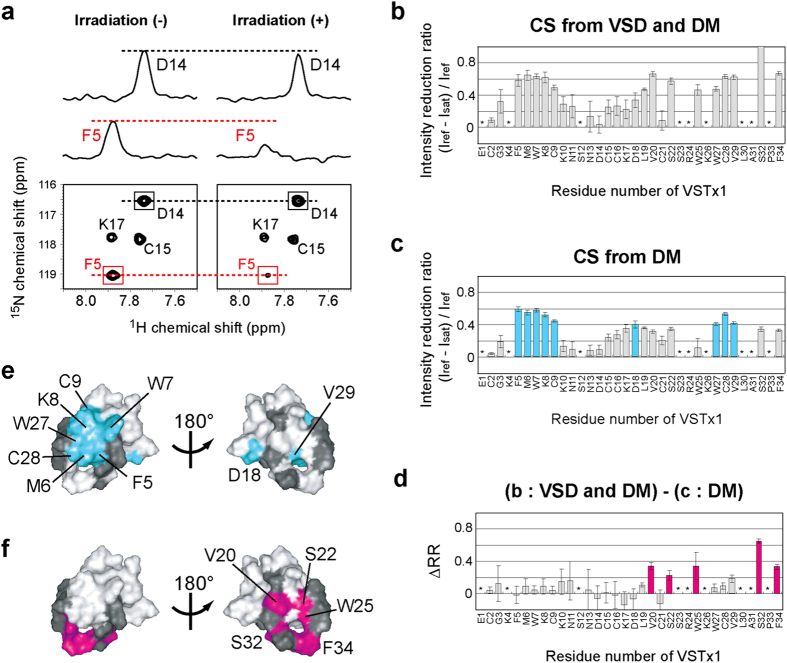
Cross saturation (CS) experiments to identify the binding sites of DM and VSD on VSTx1. (**a**) A selected potion of the ^1^H-^15^N TROSY spectra of uniformly ^2^H, ^15^N-labeled VSTx1 in complex with unlabeled VSD recorded without (left) and with (right) irradiation of the radio frequency (R.F.) pulses. Cross-sections are also shown for the signals from Phe 5 and Asp 14. (**b**) CS experiment using unlabeled VSD, where CS from DM and VSD was simultaneously observed. The ratios of the signal intensities with and without irradiation were plotted against the residue numbers of VSTx1. The residues indicated by asterisks are those with the signal-to-noise ratios less than 10. The error bars were calculated based on the signal-to-noise ratios in (**b**–**d**). (**c**) CS experiment using uniformly ^2^H-labeled VSD, where CS only from DM was observed. Bars for the residues with significant intensity reductions (minimum values of intensity reduction ratios >0.35) are colored cyan. (**d**) Plots of the difference in the intensity reduction ratios (ΔRR) in the presence of unlabeled and uniformly ^2^H-labeled VSD. Bars for the residues with significant intensity reductions (minimum values of ΔRR > 0.15) are colored magenta. (**e**,**f**) Mapping of the affected residues in (**c**,**d**) on the VSTx1 structure (PDB code 1S6X). The residues with no data are colored black.

The CS method uses uniformly ^2^H, ^15^N-labeled VSTx1 in complex with unlabeled VSD in the DM micelles. Irradiation of the radio frequency pulses on ^1^H resonances at 0 to 3.0 ppm saturates the DM and VSD resonances simultaneously. Through the dipolar-dipolar interactions, the saturation causes the signal intensity reduction of the ^1^H-^15^N TROSY signals of the VSTx1 residues located within 5 to 7 Å from the bound DM and/or VSD molecules (Fig. 4a,b).

Then, a control experiment was carried out by using uniformly ^2^H-labeled VSD instead of the unlabeled one, in which the CS only from the DM molecules should be observed. Large intensity reductions were observed for Phe 5, Met 6, Trp 7, Lys 8, Cys 9, Asp 18, Trp 27, Cys 28 and Val 29 (Fig. 4c). These residues are clustered on the structure of VSTx1, indicating that this site is the DM binding site of VSTx1 in the VSTx1-VSD complex (Fig. 4e).

In order to evaluate the CS from VSD, the intensity reduction ratios obtained by using uniformly ^2^H-labeled VSD are subtracted from those obtained by using unlabeled VSD (Fig. 4d). The differences in the reduction ratios, ΔRR, were significantly large for Val 20, Ser 22, Trp 25, Ser 32 and Phe 34, which are clustered on the opposite surface of the DM binding residues (Fig. 4f). Therefore, we concluded that the surface formed by these residues is the VSD binding site.

### Intermolecular proximal residue pairs identified by the ASCS method

The ASCS method uses amino acid selectively ^1^H-labeled VSD in a ^2^H-background instead of unlabeled VSD in the CS method. Single ASCS experiment provides the information that cross-saturated VSTx1 residue(s) is proximal to any of the ^1^H-labeled amino acid residues of VSD. When multiple ASCS results using a number of differently labeled VSD are collected, combinatorial analyses can specify pairs of the CS-source residue(s) of VSD and the corresponding CS-acceptor residue(s) of VSTx1 based on their spatial complementarities on the protein surfaces^31^.

**Figure 5:**
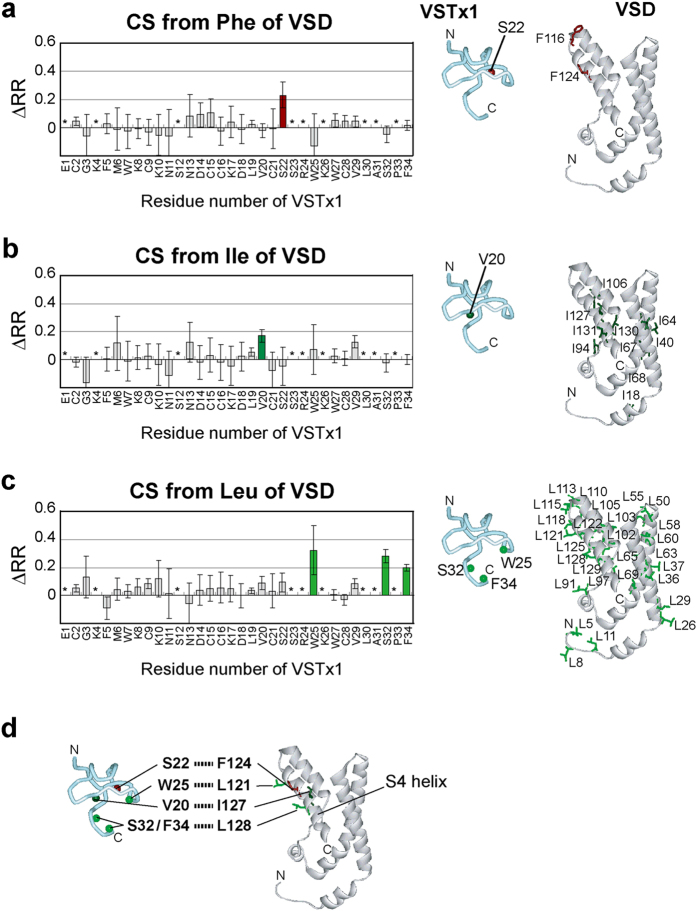
ASCS experiments to identify intermolecular proximal residue pairs. (**a**–**c**) Phe-, Ile- and Leu-selective CS experiments. The difference in the intensity reduction ratios (ΔRR) in the presence of Phe, Ile or Leu selectively ^1^H-labeled in a ^2^H-background and uniformly ^2^H-labeled VSD were plotted against the residue numbers of VSTx1, respectively (left). The residues indicated by asterisks are those with the signal-to-noise ratios less than 10. The error bars were calculated based on the signal-to-noise ratios. Bars corresponding to the residues with significant intensity reductions (minimum values of ΔRR > 0.1) are colored. The backbone amide hydrogen atoms of the affected residues are shown as balls in the VSTx1 structure (PDB code 1S6X) and the candidates of the CS-source residues are shown as sticks in the VSD structure (PDB code 2KYH) (right). (**d**) Proximal residue pairs connected by dotted lines.

We carried out three ASCS experiments, in which either Phe, Ile or Leu of VSD was selectively ^1^H-labeled in a ^2^H-background. The intensity reduction ratios of a control experiment using uniformly ^2^H-labeled VSD (Fig. 4c) were subtracted from those from each ASCS results. As a result, Ser 22 of VSTx1 was cross-saturated from [^1^H-Phe] VSD, Val 20 from [^1^H-Ile] VSD, and Trp 25, Ser 32 and Phe 34 from [^1^H-Leu] VSD (Fig. 5a–c left).

These ASCS results, with reference to the individual structures of VSTx1 and VSD, can identify the VSTx1 binding residues of VSD. Since VSD possesses only two Phe residues, Phe 116 and Phe 124, either or both of these Phe residues should be close proximity to the VSTx1 residue that is cross-saturated from [^1^H-Phe] VSD, Ser 22. Similarly, either or some of 10 Ile residues of VSD should be close to Val 20 of VSTx1, and either or some of 28 Leu residues should be close to Trp 25, Ser 32 and Phe 34 of VSTx1, respectively (Fig. 5a–c right).

Systematically, all possible combinations of the CS-source residues were evaluated based on the maximum deviation of the CS-source residues and the cross-saturated amide hydrogen atoms (Supplementary Table S2). As a result, we identified five proximal residue pairs between VSTx1 and VSD, Val 20—Ile 127, Ser 22—Phe 124, Trp 25—Leu 121, Ser 32—Leu 128 and Phe 34—Leu 128 (Fig. 5d). All the identified VSD residues (Leu 121, Phe 124, Ile 127 and Leu 128) lie on the S4 helix, indicating that VSTx1 binds to S4 of VSD.

### NMR-derived docking model of the VSTx1-VSD complex

**Figure 6:**
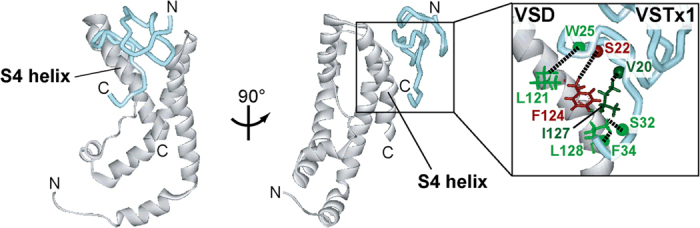
Structural model of the VSTx1-VSD complex satisfying the ASCS results. VSTx1 and VSD are depicted by a cyan tube and a white ribbon diagrams, respectively. Their binding interface is enlarged, in which the most proximal hydrogen atoms reflecting the ASCS results are connected by dotted lines.

Then, we used a docking software, HADDOCK^37^, to build a docking model of the VSTx1-VSD complex that satisfies all the proximal residue pairs experimentally identified by the ASCS method. The obtained structure exhibited no significant violation or increase of energy values (Supplementary Table S3) and all the constraints obtained by the ASCS method were satisfied in the generated structure as below; Val 20 H^N^—Ile 127 Hδ_1_ : 1.9 Å, Ser 22 H^N^—Phe 124 Hβ : 4.7 Å, Trp 25 H^N^—Leu 121 Hδ_1_ : 4.8 Å, Ser 32 H^N^—Leu 128 Hδ_2_ : 1.7 Å, and Phe 34 H^N^—Leu 128 Hδ_1_: 1.9 Å (Fig. 6). Therefore, we concluded that the structure of VSTx1-VSD complex was appropriately generated by using a docking with the NMR-derived structural constraints.

## Discussion

Ruta & MacKinnon indicated that VSTx1 binding residues exclusively reside on the VSD and the pore domain is not involved^27^. This study characterized the direct interaction between VSTx1 and the isolated VSD by fluorescence, ITC and NMR studies, indicating that VSTx1 binds to the VSD in an up conformation in lipid bilayer and DM micelles, which is consistent with the previous electrophysiological results^28^. The dissociation constant (*K*_d_) between VSTx1 and the VSD in the DM micelles was 1.5 μM, which is about three orders of magnitude larger than the previous observations in the membrane^2^,^17^. The high affinity binding of VSTx1 and the VSD in membrane consists of two effects: partitioning of VSTx1 into the lipid bilayer (membrane-water partition coefficient of VSTx1 is reported to be at most 10^5^)^17^ and the direct binding of VSTx1 and the VSD. Our ITC study used the VSD and VSTx1 that are pre-reconstituted in detergent micelles. Therefore, our ITC experiments in detergent cannot reproduce membrane partitioning effect, which probably resulted in the lower affinity than the reported one.

The DM interacting residues of VSTx1, which were identified by the CS experiment, form the hydrophobic patch on the molecular surface of VSTx1, whereas the VSD binding residues of VSTx1 (Val 20, Ser 22, Trp 25, Ser 32 and Phe 34) lie on the surface opposite to the hydrophobic patch. The VSD residues that directly interact with VSTx1 were identified by the ASCS method as Leu 121, Phe 124, Ile 127 and Leu 128, which lie in the S4 helix. The successful building of a docking model that satisfies the NMR-derived information indicates that the identified interacting surfaces possess complementary shapes, supporting the validity of the identified residues involved in the intermolecular interactions. The binding residues of VSD identified here are consistent with recent mutagenesis data^23^, but different from the alanine scanning mutagenesis of the K_v_2.1 chimera channel containing S3b and S4 from K_v_AP^38^. This inconsistency might reflect the difference between the isolated VSD and full-length K_v_AP channel although it is known that VSD contains the sole determinants for binding of VSTx1^27^. Another possibility is that the surrounding environment (detergent versus lipid molecules) of VSD would be linked to the mode of interaction of VSTx1, as described in the previous report, suggesting that the lipids play a key role in the VSD-toxin interaction^23^. It should be considered, however, that the effects of mutagenesis could be indirect, and the binding residues of VSD identified here could be occluded due to the difference in the structure and orientation to the pore domain of VSD between the K_v_2.1 chimera channel and intact K_v_AP^39^.

**Figure 7:**
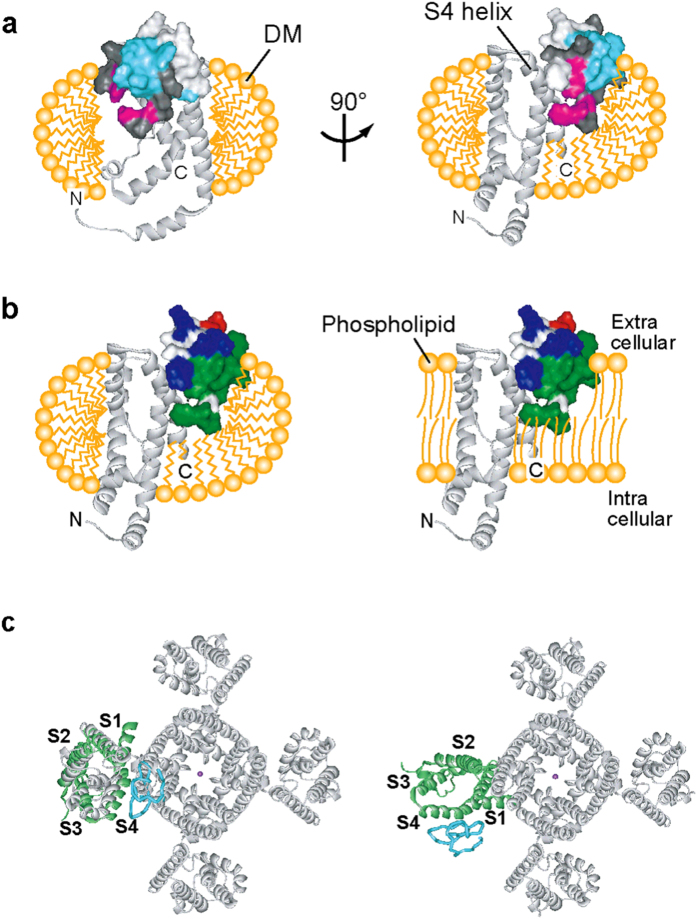
VSTx1-VSD complex in the physiological environment. (**a**) Schematic representation of the DM molecules surrounding the VSTx1-VSD complex, where the acyl chains of the DM molecules interact with the transmembrane region of VSD. The residues of VSTx1 cross-saturated from DM and VSD are colored cyan and magenta, respectively, and those with no data are colored black. (**b**) Comparison of the supposed environments of the VSTx1-VSD complex in DM micelles and phospholipid bilayer. Surface properties of VSTx1 are shown, where the acidic, basic and hydrophobic residues are colored red, blue and green, respectively. (**c**) VSTx1 binding surface in a tetrameric K_v_ channel. VSTx1-VSD complex model is superimposed to the crystal structure of K_v_1.2–K_v_2.1 chimera channel (PDB code 2R9R) (left), and then one VSD molecule of K_v_1.2–K_v_2.1 chimera channel is substituted by VSTx1-VSD complex model, followed by 70° rotation according to the previous EPR data where the arrangement of VSD and pore domain in K_v_AP is indicated to be rotated 70° to 100° relative to that of K_v_1.2–K_v_2.1 chimera^39^ (right).

The VSTx1 residues in the hydrophobic patch, which interact with DM, are exposed in the complex with VSD and thus the DM molecules would surround the complex as illustrated in Fig. 7a, where the polar residues of VSTx1 in the periphery of the hydrophobic patch lie in the extracellular side (Fig. 7b left). Therefore, in the lipid bilayer, the hydrophobic patch and peripheral polar residues of VSTx1 would contribute to the interactions with acyl and polar head groups of phospholipid molecules (Fig. 7b right), which is consistent with the notion that VSTx1 partitions at the outer leaflet of the membrane^17^,^24^,^25^. This schematic view in the membrane is similar to the structural model from the multiscale molecular dynamics (MD) simulations using palmitoyl-oleoyl phosphatidylcholine (POPC) membrane^40^. By comparing the residues of VSD that were identified in the ASCS experiments with those that showed significant toxin/VSD contacts during the MD simulations, two consensus residues in S4 (F124 and L125) are indicated, but the orientation of VSTx1 to S4 is slightly different and the C-terminus of S1 and the S1–S2 linker of VSD were also involved in the binding site of VSTx1 in the MD simulations. Although this difference might be due to the surrounding environment (DM versus POPC molecules) of VSD, both interaction modes would be physiologically acceptable, since VSTx1 partitioning into the membrane^17^,^21^,^23^,^25^ could bind to VSD from different directions. Each structural model might capture one of the most stable conformations in the presence of the DM and POPC molecules, respectively.

Figure 7c shows our structure of the VSTx1-VSD complex superimposed onto the K_v_1.2–K_v_2.1 chimera^41^, indicating that there is apparently no room around the S4 of VSD for the binding of gating modifier toxins since S4 faces to the pore domain (Fig. 7c left). However, previous EPR data indicated that the arrangement of VSD and pore domain in K_v_AP is different from that of K_v_1.2–K_v_2.1 chimera, and that VSD in K_v_AP interacts with pore domain via S1 and S2^39^. Thus, in the structure of K_v_AP, the VSTx1 binding surface on S4 seems surrounded by lipid molecules in the absence of VSTx1, and VSTx1 can bind to the S4 of VSD with the hydrophobic patch facing to the lipid side without steric hindrance with the pore domain (Fig. 7c right).

**Figure 8:**
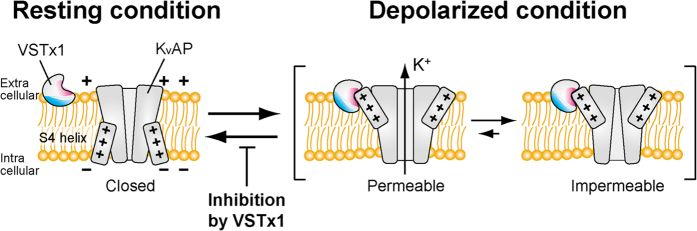
Schematic representation of the inhibitory mechanism of K_v_AP by VSTx1. K_v_AP is illustrated by a pore domain accompanied by two S4, in which voltage sensing Arg residues are depicted by “+”. VSD and DM binding sites on VSTx1 are colored magenta and cyan, respectively.

Under the resting membrane potential, VSD adopts a down conformation, which keeps the channel closed. Upon depolarization, K_v_ is activated by changing the VSD conformation from down to up, and then spontaneously inactivated by decreasing population of K^+^-permeable state and increasing that of impermeable state^42^,^43^,^44^. Upon repolarization, K_v_ returns to the resting conformation. The present results provide the inhibitory mechanism of K_v_AP by VSTx1 (Fig. 8). Under the resting membrane potential, VSTx1 is supposed to sit on the outer leaflet of the cell membrane with the hydrophobic patch buried in the membrane. Upon depolarization, where VSD adopts an up conformation with a part of the S4 helix exposed to extracellular side, the extracellular part of S4 is recognized by VSTx1. By stabilizing the up conformation of VSD, VSTx1 would delay its return to the down conformation upon repolarization and thus prolong the inactivation of K_v_AP. This inhibitory mechanism is consistent with the recent electrophysiological data^28^. Actually, the binding residues and mechanism of action of the gating modifier toxins, as well as the key elements in the VSD are inconsistent in several reports^11^,^18^,^20^,^22^,^26^,^45^,^46^,^47^, and it would be reasonable to consider that the gating modifier toxins differ in their binding surfaces, critical residues on VSD, and inhibitory mechanisms individually. Our results, however, provide important insights into one of the mechanisms of action of the gating modifier toxins on K_v_ channels.

## Methods

### Expression and purification of VSTx1

The DNA encoding VSTx1 was inserted into the pET-30 Xa/LIC plasmid (Novagen) with a Factor Xa cleavage site between an N-terminal hexahistidine tag and VSTx1 sequences. VSTx1 was expressed in *E. coli* BL21 (DE3) cells. The uniformly ^15^N- and ^13^C, ^15^N-labeled samples were prepared by growing the transformants in M9 minimal medium containing ^15^NH_4_Cl, glucose or [^13^C] glucose and [^15^N] or [^13^C, ^15^N] Celtone® base powder, respectively. Similarly, the uniformly ^2^H, ^15^N-labeled samples were prepared by growing the transformants in M9 minimal medium containing 99% ^2^H_2_O, ^15^NH_4_Cl, [^2^H] glucose and [^2^H, ^15^N] Celtone® base powder. The cells were grown at 37 °C to an A_600_ of 0.6 to 0.8 and protein expression was induced with 1.0 mM isopropyl ß-D-1-thiogalactopyranoside (IPTG) for 6 h at 37 °C. The cells were harvested and lysed by sonication, followed by centrifugation. The pellets were solubilized by 8.0 M urea. VSTx1 was purified from the supernatant of the centrifuged lysate by a HIS-Select® Nickel Affinity Gel (Sigma) column. The eluted VSTx1 was refolded by dialysis, followed by digestion with Factor Xa protease (Novagen). The cleaved hexahistidine tags were removed by HIS-Select® Nickel Affinity Gel column. VSTx1 was further purified by the reverse phase high performance liquid chromatography using an ODS-AM column (YMC).

### Expression and purification of VSD

The DNA encoding Met -12 to Lys 136 of K_v_AP channel with an N-terminal decahistidine tag followed by a HRV 3C protease cleavage site was inserted into the pMAL-c2X plasmid (New England Biolabs). For preventing the artificial dimerization of VSD, Cys -2 was substituted to Ser by the QuikChange® system (Stratagene). Furthermore, site-specific cysteine mutations described below were also introduced by QuikChange®. VSD was expressed in *E. coli* C41 (DE3) cells with an N-terminal maltose binding protein (MBP) and a decahistidine tag. The uniformly ^2^H- and ^2^H, ^15^N-labeled samples were prepared by growing the transformants in M9 minimal medium containing 99% ^2^H_2_O, [^2^H] glucose, NH_4_Cl or ^15^NH_4_Cl and [^2^H] or [^2^H, ^15^N] Celtone® base powder. The cells were grown at 37 °C to an A_600_ of 0.6 to 0.8 and protein expression was induced with 1.0 mM IPTG for 6 h at 37 °C. For the expression of the Phe, Ile and Leu selectively ^1^H-labeled samples, a 5-fold concentration of the target ^1^H-amino acid as compared to that of the ^2^H-amino acid included in [^2^H] Celtone® base powder was added at the same time of the addition of IPTG. The cells were harvested and lysed by sonication, followed by centrifugation. The pellets were solubilized by DM and centrifuged. VSD was then purified from the supernatant of the centrifuged lysate by HIS-Select® Nickel Affinity Gel (Sigma) column and Amylose Resin (New England Biolabs) column, followed by digestion with HRV 3C protease (Novagen). The cleaved MBP and decahistidine tags were removed by HIS-Select® Nickel Affinity Gel column. The gel filtration analysis using a Superdex 200 10/300 GL column (GE Healthcare) indicated that the purified VSD in DM was a monomer.

### Gel shift assay of VSD Cys mutants by Mal-PEG 2k

VSD Cys mutants were reduced with 5.0 mM DTT and desalted on a Micro Bio-Spin® column (Bio-Rad). The reactions were performed in a buffer containing 50 mM Tris-HCl (pH7.0), 100 mM KCl and 4 mM DM with Mal-PEG 2k (NOF) at a molar ratio of 1:20 for each protein:Mal-PEG at room temperature for 10 min. The excess Mal-PEG was removed by a Micro Bio-Spin® column before non-reducing SDS-PAGE.

### Fluorescence assay of VSD in POPE/POPG liposome

V119C mutant of VSD was reduced with 5.0 mM DTT and desalted on a Micro Bio-Spin® column. The reaction was performed in a buffer containing 50 mM Tris-HCl (pH 7.2) and 4 mM DM with monobromobimane (mBBr, Invitrogen) at a molar ratio of 1:10 for protein:mBBr at room temperature for 1 h. The excess mBBr was removed by a Micro Bio-Spin® column.

A 3:1 mixture of POPE/POPG (1-palmitoyl-2-oleoyl-sn-glycero-3-phosphoethanolamine/1-palmitoyl-2-oleoyl-sn-glycero-3-phospho-(1′-rac-glycerol), Avanti) was dried under nitrogen stream and then resuspended to 10 mg/mL in dialysis buffer containing 20 mM Hepes-KOH (pH 7.0), 150 mM KCl, 10% glycerol and 0.2 mM ethylenediamine tetraacetic acid (EDTA). The lipid mixture was sonicated in a bath sonicator. DM was added to the lipid mixture to 20 mM and the mixture was rotated at room temperature for 1 h. mBBr-labeled VSD was added to the lipid mixture at a weight ratio of 1:200 for protein:lipid, rotated at room temperature for 2 h and then dialyzed against dialysis buffer for 3 days at 4 °C, exchanging buffer twice a day. The liposomal solution was extruded on Avanti® Mini-Extruder (Avanti) with 11 passes through a 0.1 μm Nuclepore membrane (Whatman).

The resulting proteoliposomes were diluted 20-fold into a buffer containing 20 mM Hepes-KOH (pH 7.0), 150 mM NaCl, 10% glycerol, 0.2 mM EDTA and 47.5, 15, 4.75, 1.5 or 0.475 mM KCl, resulting in the theoretical membrane potentials of 30, 60, 90, 120 or 150 mV upon addition of valinomycin (Calbiochem), respectively. Data were collected at 25 °C on a RF-5300PC spectrofluorometer (Shimadzu) in time-acquisition mode at 1 s intervals with excitation at 394 nm, emission at 470 nm and bandwidth of 5 nm. A baseline was collected for 60 s at 0 mV and then 20 nM valinomycin was added to produce the membrane potential. The increase in the fluorescence intensities immediately after the addition of valinomycin was extrapolated from the exponential fittings of the intensity decays of each experiment. For the measurement of the inhibition of the voltage-dependent conformational change of VSD, data were collected as described above after incubation with an excess amount of VSTx1 at room temperature for 1 h.

### Isothermal titration calorimetry

Binding of VSTx1 to VSD in DM micelles was characterized by isothermal titration calorimetry (ITC) using an iTC 200 MicroCalorimeter (MicroCal). VSD was placed into a dialysis membrane (molecular weight cutoff of 50 K) and dialyzed against a buffer containing 10 mM PIPES-NaOH (pH 6.5) and 4 mM DM at 4 °C for 1 day. VSTx1 was lyophilized and diluted into the external solution used for the dialysis of VSD. 36 μM VSD was titrated with 350 μM VSTx1 at 25 °C. Heats of dilution were determined by titration into the external buffer of dialysis and subtracted from the raw titration data before analysis using the MicroCal Origin software version 5.0 provided by the manufacture. A single-site binding model was assumed. The thermodynamic parameters with error values were calculated from the fitting.

### NMR analyses

NMR samples were prepared in a buffer containing 10 mM Bis-Tris (pH 6.5), 95% H_2_O and 5% ^2^H_2_O whereas a buffer containing 30% H_2_O and 70% ^2^H_2_O was used in the CS and ASCS experiments. NMR spectra were recorded at 25 °C and 45 °C on a Bruker Avance 600 spectrometer equipped with a triple axis gradient probe and a Bruker Avance 800 spectrometer equipped with a cryogenic probe. NMR data processing and analysis were performed using Topspin 2.1 (Bruker) and Sparky (T. D. Goddard and D. G. Kneller, Sparky 3, University of California, San Francisco). The error bars were based on the signal-to-noise ratio calculated by the Sparky software.

Sequential assignments of the backbone resonances of VSTx1 were established by HNCACB, CBCA(CO)NH and C(CO)NH experiments at 45 °C. Titrations of uniformly ^15^N-labeled VSTx1 with DM or unlabeled VSD were monitored by ^1^H-^15^N HSQC spectra, whereas titrations of uniformly ^2^H, ^15^N-labeled VSD with unlabeled VSTx1 were monitored by ^1^H-^15^N TROSY spectra. The CS and ASCS experiments were performed at 25 °C on a Bruker Avance 800 spectrometer using a mixture of 200 μM uniformly ^2^H, ^15^N-labeled VSTx1 and 400 μM differently labeled VSD in 50 mM DM. For the conventional CS and its negative control experiments, unlabeled and uniformly ^2^H-labeled VSD were used, respectively. The ASCS experiments were performed using the Phe, Ile and Leu selectively ^1^H-labeled VSD, respectively. Irradiation was carried out for 1.0 or 1.5 s by a WURST-2 decoupling scheme. The saturation frequency was set at 1.5 ppm and the maximum radiofrequency amplitude was set to 0.17 kHz for WURST-2. The recycling delay was set at 4.0 s. In the CS and ASCS experiments, Lys 4, Ser 12, Ser 23, Arg 24, Lys 26, Leu 30 and Ala 31 were out of analysis due to their low signal-to-noise ratios less than 10.

The CS-source residues on VSD were identified by systematically combining the ASCS results based on the spatial complementarity of the cross-saturated amide hydrogen atoms on VSTx1 and the CS-source candidates on VSD. A conformer of each of the solution structures of VSTx1 (PDB code 1S6X) and VSD (PDB code 2KYH) were used for the analysis. The coordinates of the hydrogen atoms were generated using MOLMOL 2k.2.

### Identification of the proximal residue pairs of the VSTx1-VSD complex

All possible combinations of the CS-source residues were systematically evaluated based on the maximum deviation of the CS-source residues and the cross-saturated amide hydrogen atoms^31^. The top 20 candidates of the proximal residue pairs of VSTx1 and VSD are listed according to the maximum deviations of the amide hydrogen atoms of VSTx1 and each candidate residue on VSD (Supplementary Table S2). It should be noted that the maximum deviations are not the real distances between hydrogen atoms, where the coordinates of the CS-donor residues are replaced by the center of gravity of the hydrogen atoms. The candidates proposed by the ASCS results are clustered into 3 groups. Of these groups, group 2 and 3 are not adoptable as the candidates for the proximal residue pairs due to their steric hindrances between V20 of VSTx1 and I130 on VSD (group 2) and W25 of VSTx1 and L125 or L128 on VSD (group 3), respectively. For the subsequent docking, the candidate with the smallest maximum deviation in group 1 (denoted by an asterisk in Supplementary Table S2) was utilized as the distance constraints.

### Construction of the structural model of the VSTx1-VSD complex

The structural model of VSTx1-VSD complex was generated with the HADDOCK software^37^. The distance restraints file was generated as the unambiguous distance restraints so that the cross-saturated amide hydrogen atoms on VSTx1 and the aliphatic and aromatic hydrogen atoms of the CS-source residues on VSD should be located between 1.8 to 5.0 Å, respectively. The C-terminal segment (Leu 30 to Phe 34) of VSTx1 was defined as fully flexible during the docking. For the rigid-body energy minimization, 200 structures were generated with the 50 lowest energy solutions used for subsequent semi-flexible simulated annealing and water refinement.

## Additional Information

**How to cite this article**: Ozawa, S.- *et al.* Structural basis for the inhibition of voltage-dependent K^+^ channel by gating modifier toxin. *Sci. Rep.* **5**, 14226; doi: 10.1038/srep14226 (2015).

## Supplementary Material

Supplementary Figures and Tables

## References

[b1] Hille B. Ion channels of excitable membranes, 3rd Ed. (Sinauer, Sunderland, Mass, 2001).

[b2] Jiang Y., Ruta V., Chen J., Lee A. & MacKinnon R. The principle of gating charge movement in a voltage-dependent K^+^ channel. Nature 423, 42–48 (2003). 12721619 10.1038/nature01581

[b3] Swartz K. J. Sensing voltage across lipid membranes. Nature 456, 891–897 (2008). 19092925 10.1038/nature07620 PMC2629456

[b4] Seoh S. A., Sigg D., Papazian D. M. & Bezanilla F. Voltage-sensing residues in the S2 and S4 segments of the Shaker K^+^ channel. Neuron 16, 1159–1167 (1996). 8663992 10.1016/s0896-6273(00)80142-7

[b5] Schoppa N. E., McCormack K., Tanouye M. A. & Sigworth F. J. The size of gating charge in wild-type and mutant Shaker potassium channels. Science 255, 1712–1715 (1992). 1553560 10.1126/science.1553560

[b6] Yarov-Yarovoy V., Baker D. & Catterall W. A. Voltage sensor conformations in the open and closed states in ROSETTA structural models of K^+^ channels. Proc. Natl. Acad. Sci. USA 103, 7292–7297 (2006). 16648251 10.1073/pnas.0602350103 PMC1464335

[b7] Tao X., Lee A., Limapichat W., Dougherty D. A. & MacKinnon R. A gating charge transfer center in voltage sensors. Science 328, 67–73 (2010). 20360102 10.1126/science.1185954 PMC2869078

[b8] Li Q., Wanderling S., Sompornpisut P. & Perozo E. Structural basis of lipid-driven conformational transitions in the K_v_AP voltage-sensing domain. Nat. Struct. Mol. Biol. 21, 160–166 (2014). 24413055 10.1038/nsmb.2747 PMC3946318

[b9] Li Q. *et al.* Structural mechanism of voltage-dependent gating in an isolated voltage-sensing domain. Nat. Struct. Mol. Biol. 21, 244–252 (2014). 24487958 10.1038/nsmb.2768 PMC4116111

[b10] Takeshita K. *et al.* X-ray crystal structure of voltage-gated proton channel. Nat. Struct. Mol. Biol. 21, 352–357 (2014). 24584463 10.1038/nsmb.2783

[b11] Mouhat S., Andreotti N., Jouirou B. & Sabatier J. M. Animal toxins acting on voltage-gated potassium channels. Curr. Pharm. Des. 14, 2503–2518 (2008). 18781998 10.2174/138161208785777441

[b12] Eriksson M. A. & Roux B. Modeling the structure of agitoxin in complex with the Shaker K^+^ channel: a computational approach based on experimental distance restraints extracted from thermodynamic mutant cycles. Biophys. J. 83, 2595–2609 (2002). 12414693 10.1016/S0006-3495(02)75270-3 PMC1302345

[b13] Takeuchi K. *et al.* Structural basis of the KcsA K^+^ channel and agitoxin2 pore-blocking toxin interaction by using the transferred cross-saturation method. Structure 11, 1381–1392 (2003). 14604528 10.1016/j.str.2003.10.003

[b14] Yu L. *et al.* Nuclear magnetic resonance structural studies of a potassium channel-charybdotoxin complex. Biochemistry 44, 15834–15841 (2005). 16313186 10.1021/bi051656d

[b15] Banerjee A., Lee A., Campbell E. & MacKinnon R. Structure of a pore-blocking toxin in complex with a eukaryotic voltage-dependent K^+^ channel. eLife 2, e00594 (2013). 23705070 10.7554/eLife.00594 PMC3660741

[b16] Lou K. L. *et al.* A possible molecular mechanism of hanatoxin binding-modified gating in voltage-gated K^+^ -channels. J. Mol. Recognit. 16, 392–395 (2003). 14732930 10.1002/jmr.614

[b17] Lee S. Y. & MacKinnon R. A membrane-access mechanism of ion channel inhibition by voltage sensor toxins from spider venom. Nature 430, 232–235 (2004). 15241419 10.1038/nature02632

[b18] Swartz K. J. Tarantula toxins interacting with voltage sensors in potassium channels. Toxicon 49, 213–230 (2007). 17097703 10.1016/j.toxicon.2006.09.024 PMC1839852

[b19] Takahashi H. *et al.* Solution structure of hanatoxin1, a gating modifier of voltage-dependent K^+^ channels: common surface features of gating modifier toxins. J. Mol. Biol. 297, 771–780 (2000). 10731427 10.1006/jmbi.2000.3609

[b20] Lee C. W. *et al.* Solution structure and functional characterization of SGTx1, a modifier of K_v_2.1 channel gating. Biochemistry 43, 890–897 (2004). 14744131 10.1021/bi0353373

[b21] Jung H. J. *et al.* Solution structure and lipid membrane partitioning of VSTx1, an inhibitor of the K_v_AP potassium channel. Biochemistry 44, 6015–6023 (2005). 15835890 10.1021/bi0477034

[b22] Phillips L. R. *et al.* Voltage-sensor activation with a tarantula toxin as cargo. Nature 436, 857–860 (2005). 16094370 10.1038/nature03873

[b23] Milescu M. *et al.* Interactions between lipids and voltage sensor paddles detected with tarantula toxins. Nat. Struct. Mol. Biol. 16, 1080–1085 (2009). 19783984 10.1038/nsmb.1679 PMC2782670

[b24] Jung H. H. *et al.* Structure and orientation of a voltage-sensor toxin in lipid membranes. Biophys. J. 99, 638–646 (2010). 20643084 10.1016/j.bpj.2010.04.061 PMC2905102

[b25] Mihailescu M. *et al.* Structural interactions of a voltage sensor toxin with lipid membranes. Proc. Natl. Acad. Sci. USA 111, E5463–E5470 (2014). 25453087 10.1073/pnas.1415324111 PMC4273406

[b26] Wang J. M. *et al.* Molecular surface of tarantula toxins interacting with voltage sensors in K_v_ channels. J. Gen. Physiol. 123, 455–467 (2004). 15051809 10.1085/jgp.200309005 PMC2217462

[b27] Ruta V. & MacKinnon R. Localization of the voltage-sensor toxin receptor on K_v_AP. Biochemistry 43, 10071–10079 (2004). 15287735 10.1021/bi049463y

[b28] Schmidt D., Cross S. R. & MacKinnon R. A gating model for the archeal voltage-dependent K^+^ channel K_v_AP in DPhPC and POPE:POPG decane lipid bilayers. J. Mol. Biol. 390, 902–912 (2009). 19481093 10.1016/j.jmb.2009.05.062 PMC2778279

[b29] Shimada I. *et al.* Cross-saturation and transferred cross-saturation experiments. Prog. Nucl. Magn. Reson. Spectrosc. 54, 123–140 (2009).

[b30] Nishida N. & Shimada I. An NMR method to study protein-protein interactions. Methods Mol. Biol. 757, 129–137 (2012). 21909911 10.1007/978-1-61779-166-6_10

[b31] Igarashi S., Osawa M., Takeuchi K., Ozawa S. & Shimada I. Amino acid selective cross-saturation method for identification of proximal residue pairs in a protein-protein complex. J. Am. Chem. Soc. 130, 12168–12176 (2008). 18707104 10.1021/ja804062t

[b32] Jiang Y. *et al.* X-ray structure of a voltage-dependent K^+^ channel. Nature 423, 33–41 (2003). 12721618 10.1038/nature01580

[b33] Zhang J., Feng Y. & Forgac M. Proton conduction and bafilomycin binding by the V0 domain of the coated vesicle V-ATPase. J. Biol. Chem. 269, 23518–23523 (1994). 8089118

[b34] Lee S. Y., Letts J. A. & MacKinnon R. Functional reconstitution of purified human H_v_1 H^+^ channels. J. Mol. Biol. 387, 1055–1060 (2009). 19233200 10.1016/j.jmb.2009.02.034 PMC2778278

[b35] Zheng H., Liu W., Anderson L. Y. & Jiang Q. X. Lipid-dependent gating of a voltage-gated potassium channel. Nat. Commun. 2, 250 (2011). 21427721 10.1038/ncomms1254 PMC3072105

[b36] Ruta V., Chen J. & MacKinnon R. Calibrated measurement of gating-charge arginine displacement in the K_v_AP voltage-dependent K^+^ channel. Cell 123, 463–475 (2005). 16269337 10.1016/j.cell.2005.08.041

[b37] de Vries S. J., van Dijk M. & Bonvin A. M. The HADDOCK web server for data-driven biomolecular docking. Nat. Protoc. 5, 883–897 (2010). 20431534 10.1038/nprot.2010.32

[b38] Alabi A. A., Bahamonde M. I., Jung H. J., Kim J. I. & Swartz K. J. Portability of paddle motif function and pharmacology in voltage sensors. Nature 450, 370–375 (2007). 18004375 10.1038/nature06266 PMC2709416

[b39] Chakrapani S., Cuello L. G., Cortes D. M. & Perozo E. Structural dynamics of an isolated voltage-sensor domain in a lipid bilayer. Structure 16, 398–409 (2008). 18334215 10.1016/j.str.2007.12.015 PMC2703488

[b40] Wee C. L., Gavaghan D. & Sansom M. S. Interactions between a voltage sensor and a toxin via multiscale simulations. Biophys. J. 98, 1558–1565 (2010). 20409475 10.1016/j.bpj.2009.12.4321 PMC2856169

[b41] Long S. B., Tao X., Campbell E. B. & MacKinnon R. Atomic structure of a voltage-dependent K^+^ channel in a lipid membrane-like environment. Nature 450, 376–382 (2007). 18004376 10.1038/nature06265

[b42] Gao L., Mi X., Paajanen V., Wang K. & Fan Z. Activation-coupled inactivation in the bacterial potassium channel KcsA. Proc. Natl. Acad. Sci. USA 102, 17630–17635 (2005). 16301524 10.1073/pnas.0505158102 PMC1287484

[b43] Cordero-Morales J. F. *et al.* Molecular determinants of gating at the potassium-channel selectivity filter. Nat. Struct. Mol. Biol. 13, 311–318 (2006). 16532009 10.1038/nsmb1069

[b44] Imai S., Osawa M., Takeuchi K. & Shimada I. Structural basis underlying the dual gate properties of KcsA. Proc. Natl. Acad. Sci. USA 107, 6216–6221 (2010). 20212150 10.1073/pnas.0911270107 PMC2852003

[b45] Milescu M. *et al.* Tarantula toxins interact with voltage sensors within lipid membranes. J. Gen. Physiol. 130, 497–511 (2007) 17938232 10.1085/jgp.200709869 PMC2151668

[b46] Gupta K. *et al.* Tarantula toxins use common surfaces for interacting with K_v_ and ASIC ion channels. eLife 4, e06774 (2015) 25948544 10.7554/eLife.06774 PMC4423116

[b47] Milescu M., Lee H. C., Bae C. H., Kim J. I. & Swartz K. J. Opening the shaker K^+^ channel with hanatoxin. J. Gen. Physiol. 141, 203–216 (2013). 23359283 10.1085/jgp.201210914 PMC3557313

