# Supplementary material for: Structural basis for the inhibition of voltage-dependent K+ channel by gating modifier toxin
Source: Sci Rep. 2015 Sep 18;5:14226. doi: 10.1038/srep14226 (PMC4585561; doi:10.1038/srep14226)
Supplement: Supplementary Figures and Tables [file srep14226-s1.pdf]

Structural basis for the inhibition of voltage-dependent K^+ channel by gating modifier toxin

Shin-ichiro Ozawa[1], Tomomi Kimura[1], Tomohiro Nozaki[1], Hitomi Harada[1], Ichio Shimada[1], and Masanori Osawa [1,2,*]

[1]Graduate School of Pharmaceutical Sciences, The University of Tokyo, Hongo, Bunkyo-ku, Tokyo 113-0033, Japan

[2]Keio University Faculty of Pharmacy, Shibakoen, Minato-ku, Tokyo 105-8512, Japan

*Correspondence to osawa-ms@pha.keio.ac.jp

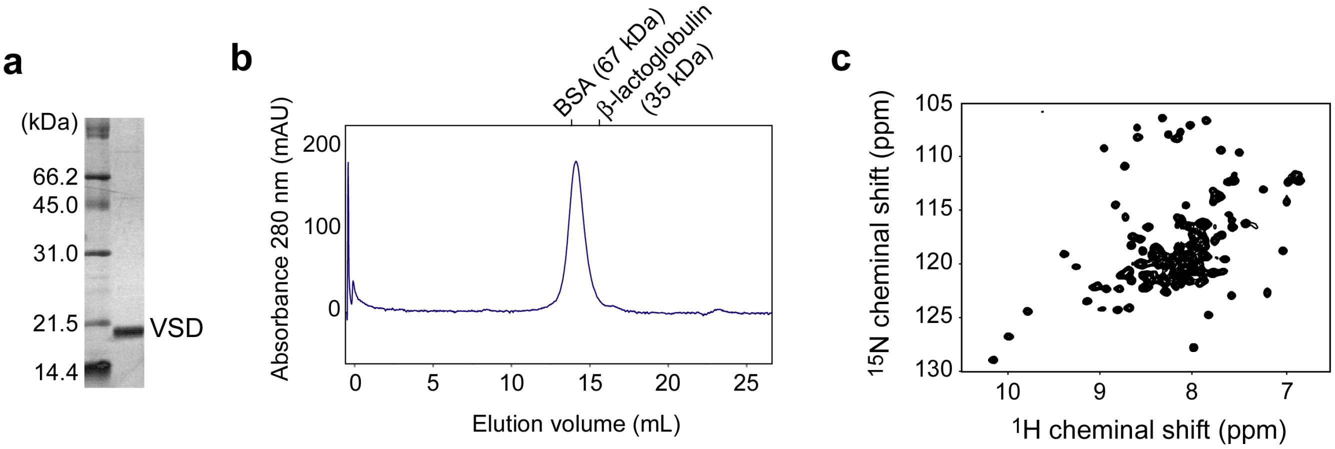

Supplementary Figure S1: Characterization of VSD.

(a) SDS-PAGE analysis of the purified VSD. (b) The gel filtration analysis of VSD solubilized in DM micelles. VSD was eluted at the elution volume corresponding to the molecular weight 60-65 kDa, which presumably consists of a VSD monomer (17 kDa) and a DM micelle (45-50 kDa). (c) ^{1}H-^{15}N TROSY spectrum of uniformly ^{2}H, ^{15}N-labeled VSD in the presence of DM.

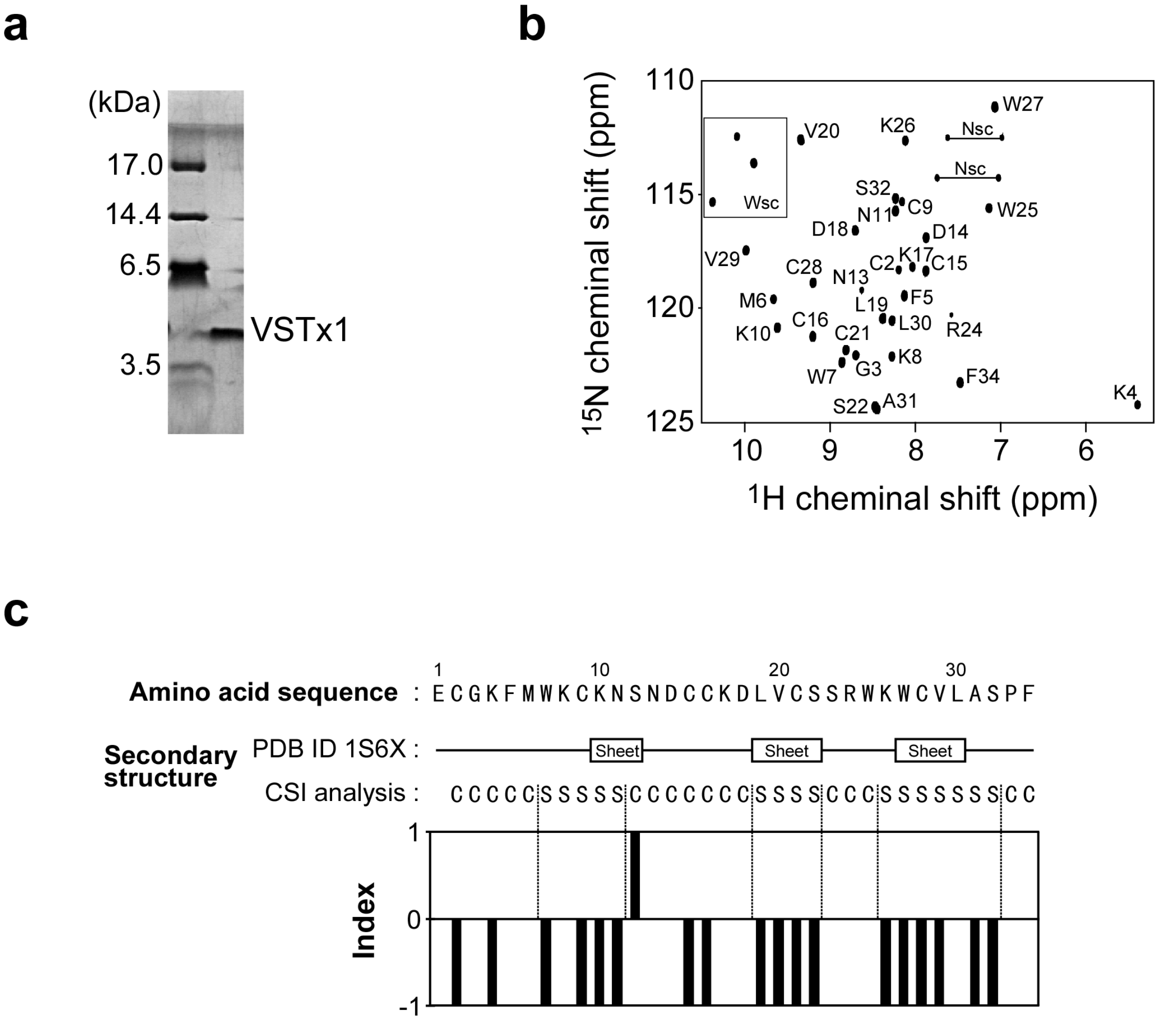

Supplementary Figure S2: Characterization of VSTx1.

(a) Tricine SDS-PAGE analysis of the purified VSTx1. (b) ^{1}H-^{15}N HSQC spectrum of uniformly ^{15}N-labeled VSTx1. “sc” indicates side chain signals. (c) The chemical shift index (CSI) of $^{13}C\alpha$ of VSTx1. The indices ‘1’ and ‘-1’ indicate the residues in which the measured chemical shift of $^{13}C\alpha$ is shifted more than 0.1 ppm upfield and downfield from the standard chemical shift, respectively. Other residues are indexed ‘0’. The secondary structure predicted from CSI values are shown by single letters, ‘C’ and ‘S’, which correspond to coil and sheet, respectively[48].

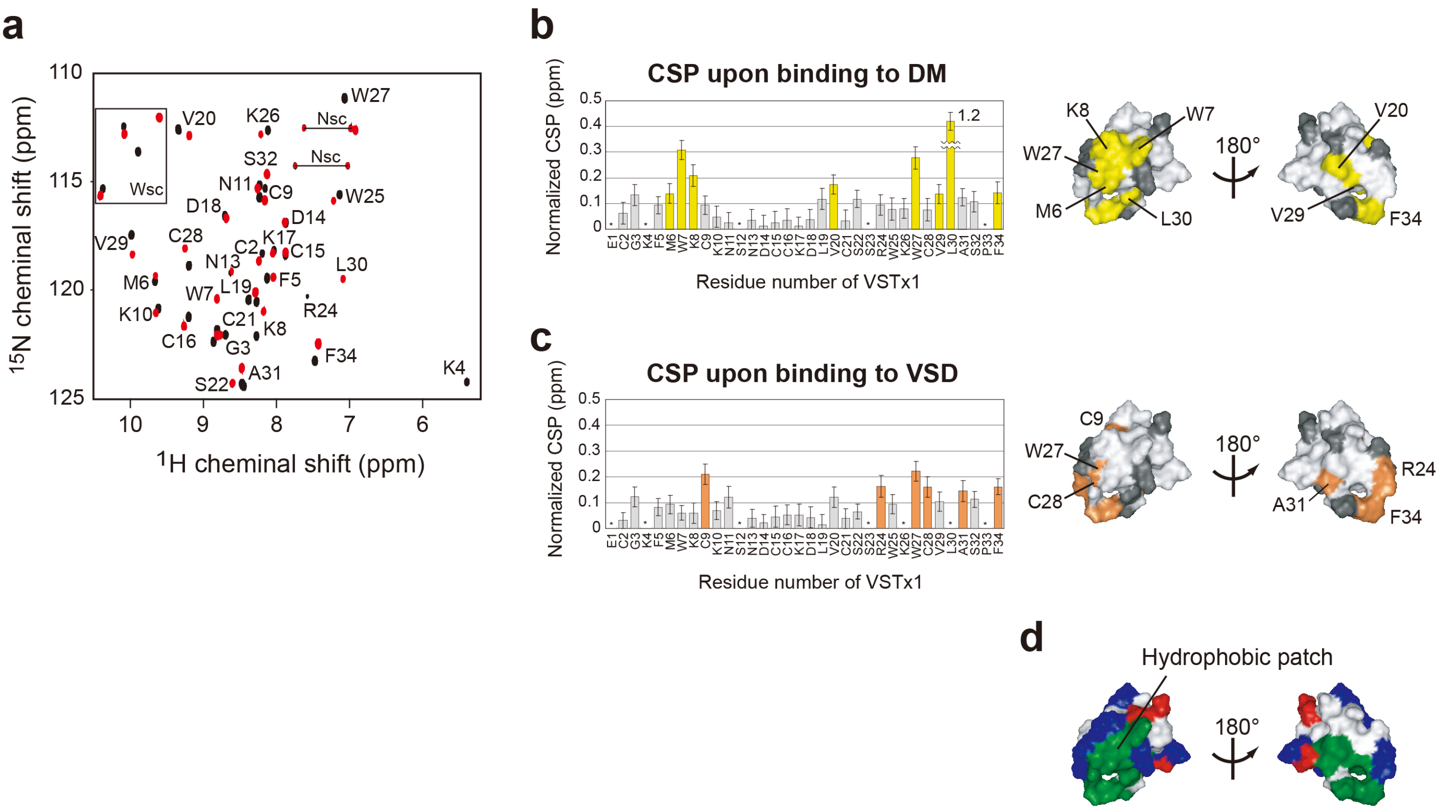

Supplementary Figure S3: Chemical shift perturbation (CSP) of the backbone amide resonances of VSTx1.

(a) ^{1}H-^{15}N HSQC spectra of uniformly ^{15}N-labeled VSTx1 in the absence (black) and presence (red) of 150 mM DM. The signal for Lys 4 disappeared upon binding to DM. (b, c) Mapping of CSP upon binding to DM (b) and VSD in the presence of 150 mM DM (c). The residues displayed more than 0.1 ppm CSP upon binding to DM and VSD are colored yellow and orange, respectively. Normalized CSP values were calculated according to the formula[49], $\{(\Delta\delta_{1H})^2 + (\Delta\delta_{15N}/6.5)^2\}^{0.5}$. Asterisks indicate the residues with no data and error bars are calculated based on the digital resolutions of the spectra. (d) Surface properties of VSTx1. The acidic, basic and hydrophobic residues are colored red, blue and green, respectively.

Supplementary Table S1: Assignments of the backbone resonances of VSTx1 at 45 °C.

Residue number	Chemical shift (ppm)			
	H	Cα	Cβ	N
C2	8.199	51.589	39.607	118.278
G3	8.713	44.472	-	107.086
K4	5.414	52.009	30.153	124.237
F5	8.132	56.759	36.570	119.467
M6	9.210	55.539	26.810	118.897
W7	8.872	55.104	26.425	122.387
K8	8.280	54.883	30.187	122.147
C9	8.249	51.006	45.110	115.748
K10	9.677	54.830	31.463	119.618
N11	8.246	49.470	38.458	115.169
S12	-	60.374	-	-
N13	8.642	52.639	35.060	119.212
D14	7.889	53.252	40.135	116.909
C15	7.889	51.006	39.293	118.349
C16	9.626	51.865	37.616	120.861
K17	8.045	56.047	30.044	118.195
D18	8.712	54.175	36.598	116.577
L19	8.399	52.373	42.131	120.46
V20	9.352	56.533	33.234	112.605
C21	8.821	52.139	37.435	121.87
S22	8.484	55.583	61.190	124.337
S23	-	58.241	60.156	-
R24	7.591	54.884	28.184	120.291
W25	7.146	53.684	26.228	115.601
K26	8.135	53.708	24.977	112.634
W27	7.085	52.647	28.276	111.151
C28	9.217	53.919	39.135	121.258
V29	9.995	57.222	33.356	117.494
L30	8.272	53.940	39.629	120.584

A31	8.457	50.380	16.838	124.475
S32	8.161	53.392	61.339	115.285
P33	-	60.985	29.272	-
F34	7.487	56.110	37.917	123.274

Supplementary Table S2: List of the CS-source candidates on VSD for the cross-saturated amide hydrogen atoms of VSTx1.

Cross-saturated amide hydrogen atoms of VSTx1	V20	S22	W25	S32	F34	Maximum deviation (Å)	Group
CS source candidates on VSD	I127	F124	L128	L125	L125	3.742	3
	I127	F124	L121	L128	L128	3.828	1*
	I127	F124	L121	L125	L125	3.834	1
	I127	F124	L121	L128	L125	3.897	1
	I130	F124	L128	L125	L125	4.333	2
	I127	F124	L128	L125	L121	4.367	3
	I131	F124	L121	L128	L125	4.444	1
	I127	F124	L125	L128	L128	4.510	3
	I131	F124	L121	L125	L125	4.579	1
	I131	F124	L121	L128	L128	4.589	1
	I127	F124	L128	L125	L125	4.648	3
	I130	F124	L128	L125	L125	4.747	2
	I131	F124	L125	L128	L128	4.832	3
	I131	F124	L128	L125	L125	4.842	3
	I127	F124	L128	L121	L121	4.901	3
	I130	F124	L121	L125	L125	4.922	2
	I130	F124	L121	L128	L128	4.936	2
	I130	F124	L128	L125	L125	4.958	2
	I131	F124	L128	L125	L125	4.968	3
	I130	F124	L121	L128	L125	4.980	2

*The residue pairs utilized in HADDOCK docking

Supplementary Table S3: HADDOCK parameters

HADDOCK score	-53.4 ± 3.9
Cluster size	48
RMSD from the overall lowest-energy structure	0.9 ± 0.7
van der Waals energy	-43.6 ± 6.4
Electrostatic energy	-44.3 ± 18.0
Desolvation energy	-1.9 ± 2.3
Restraints violation energy	9.4 ± 12.49
Buried Surface Area	1162.3 ± 78.1

References

48. Wishart, D.S. & Sykes, B.D. The ^{13}C chemical-shift index: a simple method for the identification of protein secondary structure using ^{13}C chemical-shift data. *J. Biomol. NMR* **4,** 171-180 (1994).

49. Mulder, F.A., Schipper, D., Bott, R. & Boelens, R. Altered flexibility in the substrate-binding site of related native and engineered high-alkaline Bacillus subtilisins. *J. Mol. Biol.* **292,** 111-123 (1999).
